# Improving segmentation precision in prostate cancer adaptive radiation therapy with a patient-specific network

**DOI:** 10.1371/journal.pone.0332603

**Published:** 2025-09-19

**Authors:** Joonil Hwang, Byung-Hee Kang, Younghee Park, Dong Hyeok Choi, Jin Sung Kim, Seungryong Cho, Eungman Lee

**Affiliations:** 1 Department of Nuclear and Quantum Engineering, MIRLAB, Korea Advanced Institute of Science and Technology, Daejeon, Republic of Korea; 2 Department of Radiation Oncology, Ewha Womans University College of Medicine, Seoul, Republic of Korea; 3 Department of Radiation Oncology, MPBEL, Yonsei Cancer Center, Yonsei University College of Medicine, Seoul, Republic of Korea; 4 Department of Medicine, Yonsei University College of Medicine, Seoul, Republic of Korea; Chung-Ang University Gwangmyeong Hospital, KOREA, REPUBLIC OF

## Abstract

Adaptive radiotherapy (ART) enhances prostate cancer treatment by accounting for daily anatomical variations, but clinical implementation remains limited due to the need for accurate and efficient auto segmentation; manual corrections after automated contouring often hinder workflow efficiency. To address this, we propose a patient-specific network (PSN) approach for clinical target volume (CTV) segmentation using cone-beam computed tomography (CBCT). This retrospective study included 26 prostate cancer patients treated with CBCT-guided online ART using the Ethos therapy system, comprising 119 retrospectively exported fractions. The PSN framework uses a two-stage strategy: initial pre-training followed by patient-specific fine-tuning via PSN_adaptive_ or PSN_sequence_, implemented with the Swin UNETR architecture. This approach is distinct from static personalization methods as it continuously adapts to daily anatomical changes. Segmentation performance was compared against deformable registration and generalized deep learning models using the Dice similarity coefficient (DSC), 95^th^ percentile Hausdorff distance (HD), and mean surface distance (MSD). PSN significantly improved segmentation performance, with PSN_adaptive_ achieving a DSC of 0.978 ± 0.005, HD of 1.681 ± 0.743 mm, and MSD of 0.510 ± 0.035 mm by the fifth fraction, with accuracy improving across sequential fractions. Visual assessments confirmed high agreement with physician contours, especially in anatomically complex regions. These findings support the PSN framework as a clinically feasible and accurate solution for patient-specific segmentation in prostate ART, potentially reducing the need for manual editing, streamlining workflow efficiency, and enhancing the precision of adaptive treatment delivery.

## Introduction

External beam radiotherapy plays a key role in the management of prostate cancer across all stages, and hypo-fractionated regimens are increasingly adopted for patient convenience. A recent randomized phase III trial confirmed the efficacy of stereotactic body radiotherapy (SBRT) as a curative treatment for localized prostate cancer [1]. However, the study also reported a higher incidence of late genitourinary toxicity with SBRT, emphasizing the importance of precise treatment delivery and effective normal tissue sparing, particularly in high-dose per-fraction regimens. Adaptive radiotherapy (ART) has emerged as a strategy to address daily anatomical variations and to improve the therapeutic ratio further in prostate cancer [2,3]. By adjusting the treatment plan to daily changes in prostate and organ-at-risk (OAR) positioning, ART facilitates smaller margins and improved dose conformity. Planning studies have shown that daily ART can significantly reduce OAR doses, thereby potentially lowering complication risks [2].

Clinical studies on the organ motion consistently demonstrate that the inter‑fraction geometric variation can exceed the 5 mm isotropic margin conventionally used in prostate RT. Langen et al. analyzed 550 fractions with electromagnetic tracking and found that the prostate spent a median 13.6% of beam‑on time with a variation of ≥ 3 mm from its planning position and 3.3% with ≥ 5 mm; in some fractions the variation of ≥ 3 mm threshold persisted for 99% of the treatment time [4]. Ghilezan et al. showed with cine‑MRI that rectal filling drives rapid posterior shifts, with ≥ 3 mm displacement reached within 60 s in 10% of full‑rectum scans [5]. For the seminal vesicles, a recent systematic review reported inter‑fraction systematic/random errors of 1–7 mm and 1–5 mm, respectively, with median centroid shifts around 4 mm, recommending ≥ 8 mm planning margins [6]. Shape‑based (deformable) changes of 2–4 mm have also been observed on serial cone-beam computed tomography (CBCT) registrations [7]. These magnitudes of variation clearly exceed conventional margins, especially in hypo-fractionated SBRT, and strongly motivate the use of daily online ART.

Early clinical data have also shown that adaptive SBRT reduces acute urinary toxicity by 44% and bowel toxicity by 60% compared to non-adaptive SBRT, reinforcing its potential to enhance treatment tolerability and outcomes [8]. Despite these advantages, the clinical implementation of ART remains challenging. The requirement for daily target re-contouring and plan re-optimization increases both the workload and treatment time, limiting the adoption of real-time ART in routine clinical practice. Among these challenges, efficient and accurate auto-segmentation stands out as one of the most critical barriers to routine clinical implementation of ART. Recent clinical evaluations using the Ethos platform in breast cancer have demonstrated the feasibility of ART workflows and auto-segmentation performance, supporting their potential in streamlining adaptive radiotherapy across disease sites [9,10]. Therefore, efficient automation is essential to streamline workflows and fully integrate ART into prostate cancer radiotherapy (RT).

Recent advancements in artificial intelligence (AI), particularly deep-learning-based auto segmentation (DLAS), have enabled the highly accurate segmentation of various anatomical structures [11]. In prostate cancer radiotherapy planning, DLAS achieves high geometric accuracy (DSC 0.83–0.92), with most AI-generated contours deemed clinically acceptable [12–15]. However, training DLAS typically requires large, consistently contoured datasets, posing challenges for developing patient-specific auto-contouring models tailored to individual risk profiles. When delineating the prostate clinical target volume (CTV) for definitive RT, the extent of seminal vesicle (SV) inclusion varies according to the patient risk group, and currently, no clear consensus exists regarding the precise extent of SV coverage [16,17]. Similarly, CTV delineation for post-operative RT is even more challenging because the target is a virtual volume intended to encompass potential microscopic tumor cells rather than a visible tumor. Contouring inherently involves physician judgment, complicating standardization and limiting the applicability of general auto-contouring models [18,19]. In daily adaptive ART, the main challenge of auto-contouring is not to redefine the CTV daily based on patient risk, but to maintain the initial contour in response to inter-fractional variations, thereby ensuring consistent treatment accuracy and reproducibility.

Previously, we evaluated the utility of a patient-specific network (PSN), a novel deep-learning (DL) framework that utilizes the entire daily CBCT sequence throughout the treatment course. PSN is designed to ensure robust segmentation despite anatomical changes during the treatment period by adapting to daily variations, making it an effective solution for adaptive ART environments. The PSN model is not merely personalized but adaptive, providing a solution that can be directly applied to improve segmentation in ART.

This study aimed to develop a DL algorithm for accurate CTV segmentation using daily CBCT scans of patients with prostate cancer who underwent online ART. Specifically, we evaluated whether a PSN model offers advantages compared to a generalized DL model and deformable image registration (DIR).

## Materials and methods

### Patient data

This retrospective study analyzed data from 26 patients with prostate cancer who underwent CBCT-guided online ART using the Ethos therapy system (Varian Medical Systems, Palo Alto, CA) at Ewha Womans University Seoul Hospital between November 2022 and June 2024. The study was approved by the Institutional Review Board of Ewha Womans University Seoul Hospital (approval number: 2024-03-012-002), and all procedures were conducted according to applicable guidelines and regulations. De-identified clinical data were accessed for research purposes on 15 July 2024. All data were anonymized prior to analysis, and the researchers did not have access to any personally identifiable information. All patients were treated with definitive intent. Among eleven high-risk patients, conventional fractionated whole pelvic radiotherapy (WPRT) of 46 Gy or 50 Gy was initially administered without adaptation, followed by online ART for a prostate boost. For these prostate boost sessions, eight patients received a hypo-fractionated regimen (18 Gy in three to four fractions), while the remaining patients underwent conventionally fractionated prostate boosts (24–34 Gy in 12–17 fractions). The other 15 patients underwent prostate SBRT at a dose of 36.25 Gy in five fractions. WPRT CBCTs were excluded because their clinical target volume (pelvic nodes ± prostate) differs fundamentally from the prostate‑only CTV addressed in this study and including them would have required a separate segmentation and dosimetric framework beyond our scope. After this exclusion, the final dataset comprised 119 retrospectively exported daily CBCT fractions: 75 SBRT, 29 hypofractionated‑boost and 15 conventional‑boost fractions. For each patient, we analyzed the earliest adaptive CBCTs, up to a maximum of five fractions: SBRT courses contributed all five fractions; hypofractionated boosts contributed all available three or four fractions; conventional boosts contributed the first five fractions in chronological order, thereby capping every regimen at five training cases and emulating an early‑course adaptation scenario. Detailed patient characteristics are provided in S1 Table in S1 File.

### Models

The PSN framework utilized the Swin UNETR architecture, which combines transformer-based and convolutional components for CTV segmentation [20]. Swin UNETR is implemented natively in MONAI v1.3 [21] and has demonstrated strong performance in brain-tumor, abdominal-organ, and prostate segmentation tasks [20]. Recent advances, such as Swin-Transformer pre-training for 3-D medical images [22] and scalable depth/width variants [23], further support their suitability for volumetric radiotherapy workflows.

**Fig 1:**
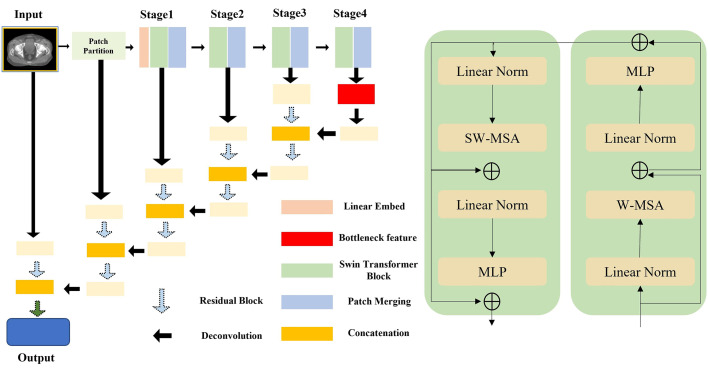
Schematic illustration of the Swin UNETR architecture used for volumetric segmentation of the prostate CTV.

Fig 1 schematically depicts the encoder–decoder: four Swin-Transformer stages extract multi-scale representations, and a symmetric decoder reconstructs high-resolution masks via skip concatenations.

For full reproducibility, the Swin UNETR was configured as follows. CBCT volumes were partitioned into non-overlapping patches of size 2 × 2 × 2 voxels and linearly projected into an embedding space with dimension 48. The encoder consisted of four Swin Transformer stages, each containing 2 transformer blocks (total 8 layers). The number of feature channels doubled at each stage, progressing as 48 → 96 → 192 → 384, with attention heads configured as 3, 6, 12, and 24, respectively. Self-attention computations were restricted to 7 × 7 × 7 voxel windows, employing shifted windowing for subsequent layers. Patch merging at the end of each stage reduced spatial resolution by a factor of two, aggregating the stage-4 features into a bottleneck with 768 channels.

The decoder adopted a symmetric U-shaped structure, using residual blocks comprising two 3 × 3 × 3 convolutional layers with InstanceNorm3D normalization. Features were up sampled using 3D transposed convolutions (stride = 2) and concatenated with corresponding encoder features at each resolution via skip connections. A drop-path rate of 0.10 was employed for regularization. The final segmentation output was generated by a 1 × 1 × 1 convolutional layer with SoftMax activation for binary classification (CTV vs. background). All codes ran under PyTorch 1.12 with MONAI v1.3.

### PSN framework

The dataset was split patient-wise: 21 patients (94 fractions) formed the pre-training cohort, while the remaining five patients (25 fractions) were reserved for sequential fine-tuning and testing. Stratified randomization preserved the SBRT-to-boost ratio across the two subsets. The innovative PSN framework constructs patient-specific DL models using a two-stage training strategy tailored to personalized medical applications. In the first stage, DL models were trained on CBCT scans from 21 patients, with performance outcomes assessed on the first to fifth fractions of CBCT scans from five remaining patients. The second stage introduced two specific methodologies: PSN_adaptive_ and PSN_sequence_. These approaches systematically trained and evaluated the pre-trained network on sequential fractions of each patient.

**Fig 2:**
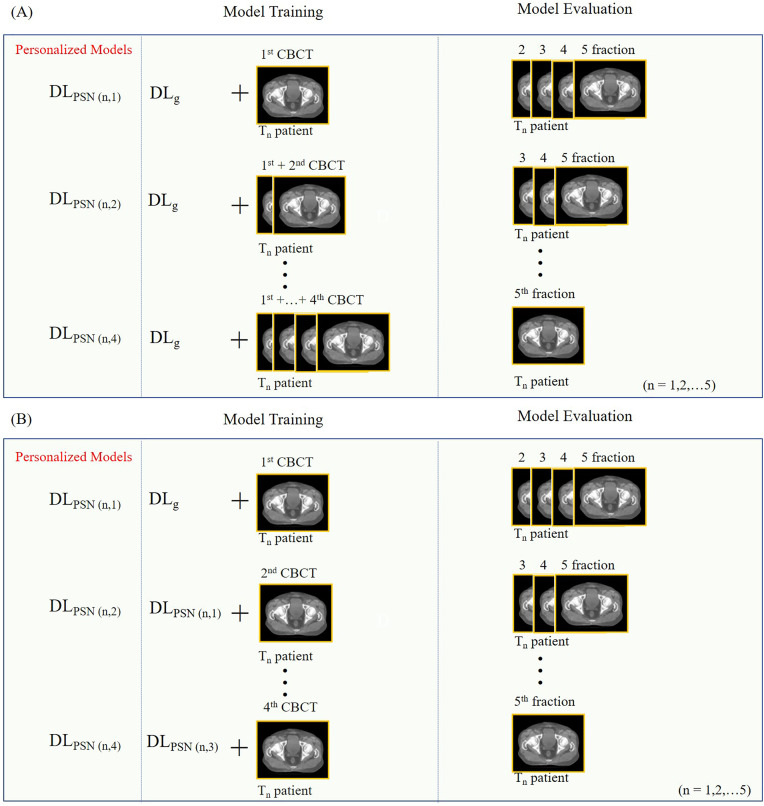
Training workflows for the PSN framework. (a) PSN_adaptive_: the model is fine-tuned on cumulatively increasing fractions and tested on subsequent ones. (b) PSN_sequence_: the model is sequentially fine-tuned using the previously trained weights for each next fraction. DL_g_ represents the pre-trained model, and n denotes the patient index (n = 1, 2,..., 5).

In PSN_adaptive_ (Fig 2(a)), the network is incrementally fine-tuned. Training begins with the first fraction, and testing is conducted on the second to fifth fractions. The model is then fine-tuned using the first and second fractions and tested on the third to fifth fractions. This pattern continues until the model is fine-tuned with the first to fourth fractions and tested on the fifth fraction. As more fractions are added to the training set, the time required for fine-tuning increases, as the network is continually updated with additional data. This approach is designed to improve performance as more patient-specific data is used for training, but the trade-off is longer training times as more fractions are incorporated.

By contrast, PSN_sequence_ (Fig 2(b)), is explicitly framed as a continual learning progression: each update uses the most recently adapted model and the next new fraction, sequentially propagating knowledge forward while keeping the amount of new data per step fixed. Because every adaptation step involves the same-sized input (one fraction), the per-step training time remains constant, making PSN_sequence_ a more time-efficient mechanism for patient-specific refinement while still leveraging previously acquired patient information.

### Preprocessing and data augmentation

To achieve accurate segmentation of the prostate CTV, we employed multiple neural networks and automated segmentation techniques, supported by a series of preprocessing and augmentation strategies designed to enhance model performance. Preprocessing included pixel intensity normalization, resolution standardization, and spatial augmentations (random cropping, flipping, and rotation). Specifically, CBCT intensities were clipped to a range of −350 to +350 HU to ensure consistency across the dataset. The resolution of all scans was standardized to 1.0 × 1.0 × 2.0 mm to maintain uniformity during the model training process.

To expand the dataset and improve the model’s robustness, random augmentations were applied to the CBCT scans. Each scan was randomly cropped to a size of 96 × 96 × 96, with flipping and rotation performed along all three axes at a probability of 0.1. Additionally, intensity augmentation was introduced by randomly shifting the intensity values with a probability of 0.5 and an offset of 0.1, enhancing the model’s ability to adapt to variations in CBCT intensity profiles. The augmentation parameters were adapted from the Swin UNETR framework [21] and were empirically adjusted to account for the anatomical scale and image characteristics of prostate CBCT.

### Optimization strategy

To balance overlap accuracy with voxel-wise classification, we optimized the network with a composite loss in which the Dice component and the cross-entropy component were each assigned 50% weight (1: 1). The model was trained using the AdamW optimizer, with a learning rate of 1 × 10−4 and weight decay of 1 × 10−5. Mixed precision training with GradScaler was applied to ensure numerical stability. All experiments were performed on a workstation equipped with an NVIDIA RTX 3090 GPU (24 GB VRAM).

### Training strategy

In the first stage of the PSN framework, the network was pre-trained on CBCT scans from 21 patients over 30,000 iterations, leveraging a diverse training set to establish a robust and generalized foundation. In the second stage, the framework adopted two distinct fraction-based sequential approaches: PSN_adaptive_ and PSN_sequence_.

In PSN_adaptive_, the network was fine-tuned for 50 epochs using CBCT scans from the first fraction of each patient and subsequently tested on the second to fifth fractions. This process was iteratively expanded, with fine-tuning incorporating the first and second fractions to test on the third to fifth fractions, and continuing this pattern until the network was fine-tuned on the first to fourth fractions and tested on the fifth fraction.

In PSN_sequence_, the network was fine-tuned for 50 epochs per fraction, following a progressive fine-tuning strategy. Starting from the first fraction, the model was fine-tuned sequentially, using the previously trained weights to train and test on the next fraction. This process continued iteratively, with the network fine-tuned on the second fraction and tested on the third, then fine-tuned on the third fraction and tested on the fourth, and so forth. By saving and loading the model at each stage, the approach leveraged accumulated learning from prior fractions to refine the model’s performance, focusing on temporal continuity and personalized optimization.

We selected 50 epochs after a pilot sweep (20, 40, 50, 80 epochs) on three validation patients: validation DSC plateaued between 40 and 45 epochs, and additional training (<1 percentage-point gain) did not justify longer wall-time. 50 epochs therefore guarantee full convergence while keeping per-fraction fine-tuning under ~2 minutes on our GPU.

This dual-stage training strategy exemplifies the transition from generalization during the pre-training phase to the development of highly personalized models. By integrating both PSN_adaptive_ and PSN_sequence_, the framework achieved segmentation accuracy optimized for prostate cancer treatment planning.

### Evaluation

To assess the performance of our network, we employed three widely used metrics: the Dice similarity coefficient (DSC), the 95^th^ percentile Hausdorff distance (HD), and the mean surface distance (MSD). Detailed formulae and implementation steps are provided in S1 Appendix (Eqs. 1–3) in S1 File. Each of these metrics offers unique insights into the accuracy and reliability of segmentation results, ensuring a comprehensive evaluation of the model.

To compare segmentation methods, we analyzed paired differences in DSC, HD and MSD for the same five patients (n = 5). Owing to the small sample size, the two-sided Wilcoxon signed-rank test (α = 0.05) was used as the sole inferential test; Holm-adjusted p-values are reported. Effect sizes are expressed as rank-biserial correlations (r) with 95% bootstrap confidence intervals (10 000 resamples). Results with 0.05 < p ≤ 0.10 are described as marginally significant.

To evaluate segmentation approaches for prostate CTV, we implemented a comparative framework encompassing three distinct methods: a generalized DL model, the deformed planning CT obtained from Ethos (via deformable image registration), and the PSN model. Each method was designed to address specific challenges in relation to segmentation accuracy and adaptability, and their evaluation provided a comprehensive understanding of segmentation performance capabilities across various scenarios.

The first method employed a generalized DL model trained on CBCT scans from multiple patients. This approach prioritized generalization over personalization, leveraging a broad dataset to achieve robust segmentation across diverse cases. The model served as a baseline for evaluating segmentation accuracy without patient-specific adaptation.

The second method involved the use of deformed planning CTs generated by the Ethos system. Here, deformable image registration was applied to align the planning CT with the CBCT. The resulting deformation field was then propagated to the planning CT’s CTV, enabling the evaluation of the segmentation accuracy based on the deformed planning CT. This method incorporated patient-specific alignment but was limited by the inherent constraints of the deformable registration process.

The third method utilized the PSN framework, which employed a two-stage training process. In the first stage, a pre-trained model was developed using CBCT scans from multiple patients to establish a robust foundation. In the second stage, the PSN framework fine-tuned the model by incorporating CBCT data from sequential fractions of each patient. This deliberate overfitting process enabled the model to adapt to patient-specific anatomical variations, resulting in a highly personalized segmentation model.

For each method, segmentation accuracy was assessed by comparing the predicted volumes to reference ground truths using three key metrics: the DSC, HD, and MSD. Statistical analyses, including hypothesis testing, were conducted to compare the performance outcomes of the three methods, ensuring a rigorous evaluation of their prostate CTV segmentation capabilities. All statistical evaluations were conducted using the R programming language.

### Dosimetric evaluation

In accordance with ICRU Report 83 [24], four dose–volume indices were analyzed for the CTV. D_95_ and D_98_ denote the minimum doses received by 95% and 98% of the CTV, respectively, and serve as “near‑minimum” coverage metrics. D_mean_ is the arithmetic mean dose to the entire CTV, reflecting overall energy deposition, while D_2_ denotes the near‑maximum dose, i.e., the dose received by the hottest 2% of the CTV. These indices collectively describe under‑coverage (D_95_, D_98_), overall conformity (D_mean_) and over‑dosage (D_2_).

For every CBCT, the three‑dimensional dose distribution was recalculated with the adaptive plan generated by the Ethos system, keeping beam geometry and monitor units unchanged. The four indices were extracted from five contour sets, physician reference, DIR, the pre‑trained network, PSN_adaptive_ and PSN_sequence_ and absolute dose errors were computed as:


$$\left|\Delta D_{x}\right|=\left|D_{x}^{method}-D_{x}^{physician}\right|\;(\text{Gy})\text{ for }x\in \left\{95,98,mean,\;2\right\}$$ (1)

Paired two‑sided Wilcoxon signed‑rank tests (α = 0.05) compared PSN‑adaptive and PSN‑sequence with DIR and with the pre‑trained model across the five evaluation patients (n = 5). Holm correction controlled the family‑wise error rate, and effect sizes were expressed as rank‑biserial correlations with 95% bootstrap confidence intervals based on 10 000 resamples. All statistical analyses were performed in R.

## Results

### Evaluation results for CTV segmentation with the PSN framework

The segmentation accuracy for prostate CTV was evaluated using the DSC, HD, and MSD. The results highlight the improvements achieved by incorporating the PSN framework, leveraging CBCT data across multiple fractions for patient-specific training. Notably, both PSN_adaptive_ and PSN_sequence_ approaches demonstrated substantial performance gains compared to the deformed planning CT and pre-trained models.

### CTV results

**Fig 3:**
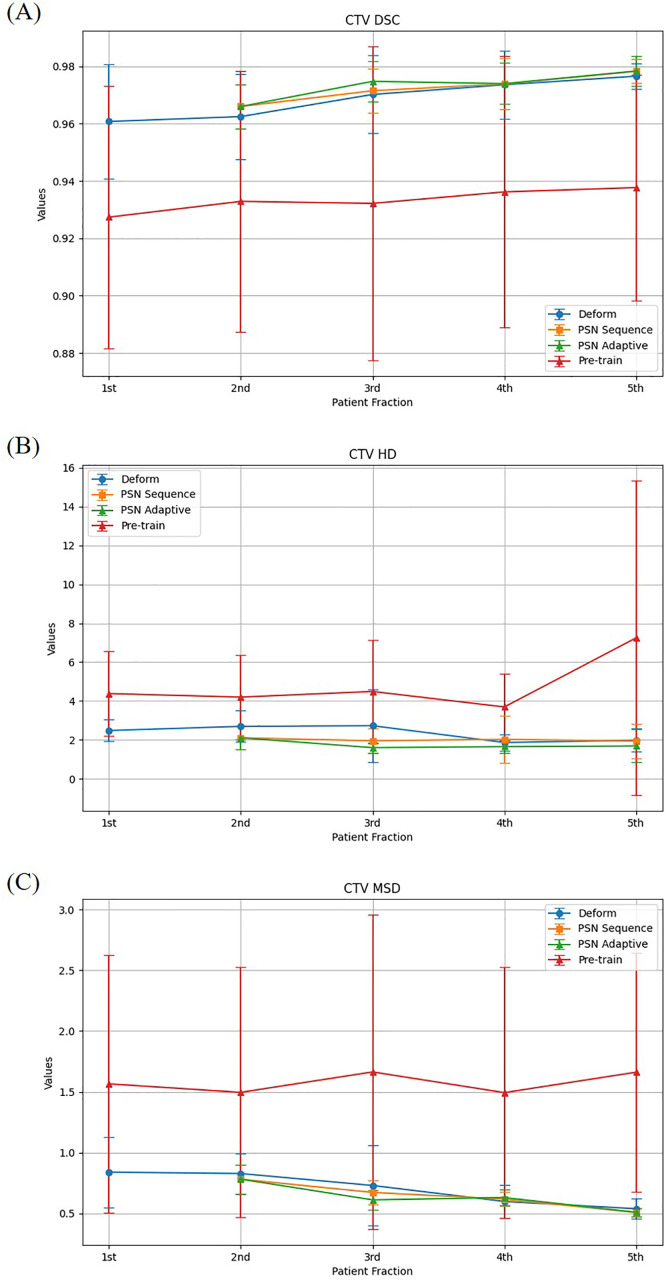
Average DSC, HD, and MSD values (± standard deviation) for prostate CTV segmentation. Results are shown for the deformable registration method (ETHOS), pre-trained model (Swin UNETR), PSN_adaptive_ (Fig 2(a)), and PSN_sequence_ (Fig 2(b)).

The progression of CTV segmentation accuracy across fractions is depicted in Tables 1 and 2, and Fig 3 compares the deformed planning CT, pre-trained model, and the two variations of the PSN framework, i.e., PSN_adaptive_ and PSN_sequence_. The performance is evaluated using DSC, HD, and MSD.

**Table 1:** Average DSC, HD, and MSD for the Deform from ETHOS, the pre-trained Swin UNETR model, and the PSN_adaptive_ approach using Swin UNETR, along with their standard deviations, as illustrated in Fig 2(a).

		Deform	Pre-train	PSN trained on 1 fx	PSN trained on 1–2 fxs	PSN trained on 1–3 fxs	PSN trained on 1–4 fxs
**CTV DSC**	**1st**	0.961 ± 0.020	0.927 ± 0.046				
	**2nd**	0.963 ± 0.015	0.933 ± 0.046	0.966 ± 0.009			
	**3rd**	0.970 ± 0.014	0.932 ± 0.055	0.972 ± 0.010	0.975 ± 0.007		
	**4th**	0.974 ± 0.012	0.936 ± 0.047	0.972 ± 0.007	0.973 ± 0.008	0.974 ± 0.007	
	**5th**	0.977 ± 0.004	0.938 ± 0.039	0.971 ± 0.008	0.975 ± 0.007	0.976 ± 0.007	0.978 ± 0.005
**CTV HD (mm)**	**1st**	2.480 ± 0.560	4.377 ± 2.193				
	**2nd**	2.694 ± 0.822	4.198 ± 2.174	2.103 ± 0.688			
	**3rd**	2.727 ± 1.868	4.487 ± 2.662	1.685 ± 0.449	1.595 ± 0.265		
	**4th**	1.860 ± 0.418	3.690 ± 1.726	1.813 ± 0.377	1.642 ± 0.359	1.649 ± 0.321	
	**5th**	1.979 ± 0.597	7.249 ± 8.094	2.141 ± 0.877	1.943 ± 1.015	1.966 ± 1.192	1.681 ± 0.847
**CTV MSD (mm)**	**1st**	0.840 ± 0.290	1.566 ± 1.057				
	**2nd**	0.828 ± 0.166	1.496 ± 1.028	0.782 ± 0.133			
	**3rd**	0.730 ± 0.328	1.664 ± 1.293	0.669 ± 0.118	0.612 ± 0.080		
	**4th**	0.599 ± 0.134	1.494 ± 1.030	0.693 ± 0.091	0.657 ± 0.098	0.631 ± 0.066	
	**5th**	0.540 ± 0.083	1.662 ± 0.984	0.678 ± 0.090	0.613 ± 0.058	0.570 ± 0.055	0.510 ± 0.035

**Table 2:** Average DSC, HD, and MSD for the PSN_adaptive_ and the PSN_sequence_ approach using Swin UNETR, along with their standard deviations, as illustrated in Fig 2.

		PSN_adaptive_				PSN_sequence_			
		Trained on 1 fx	Trained on 1–2 fxs	Trained on 1–3 fxs	Trained on 1–4 fxs	Trained on 1 fx	Trained on 2 fx	Trained on 3 fx	Trained on 4 fx
**CTV DSC**	**2nd**	0.966 ± 0.009				0.966 ± 0.009			
	**3rd**	0.972 ± 0.010	0.975 ± 0.007			0.972 ± 0.010	0.970 ± 0.009		
	**4th**	0.972 ± 0.007	0.973 ± 0.008	0.974 ± 0.007		0.972 ± 0.007	0.968 ± 0.007	0.972 ± 0.006	
	**5th**	0.971 ± 0.008	0.975 ± 0.007	0.976 ± 0.007	0.978 ± 0.005	0.971 ± 0.008	0.971 ± 0.008	0.975 ± 0.006	0.976 ± 0.005
**CTV HD (mm)**	**2nd**	2.103 ± 0.688				2.103 ± 0.688			
	**3rd**	1.685 ± 0.449	1.595 ± 0.265			1.685 ± 0.449	1.860 ± 0.418		
	**4th**	1.813 ± 0.377	1.642 ± 0.359	1.649 ± 0.321		1.813 ± 0.377	1.987 ± 0.252	1.642 ± 0.359	
	**5th**	2.141 ± 0.877	1.943 ± 1.015	1.966 ± 1.192	1.681 ± 0.847	2.141 ± 0.877	2.140 ± 0.726	1.912 ± 0.825	2.198 ± 0.845
**CTV MSD** **(mm)**	**2nd**	0.782 ± 0.133				0.782 ± 0.133			
	**3rd**	0.669 ± 0.118	0.612 ± 0.080			0.669 ± 0.118	0.730 ± 0.158		
	**4th**	0.693 ± 0.091	0.657 ± 0.098	0.631 ± 0.066		0.693 ± 0.091	0.768 ± 0.080	0.687 ± 0.095	
	**5th**	0.678 ± 0.090	0.613 ± 0.058	0.570 ± 0.055	0.510 ± 0.035	0.678 ± 0.090	0.680 ± 0.109	0.601 ± 0.065	0.546 ± 0.070

On average across fractions 1–5, the deformed planning CT achieved an average DSC of 0.974, while the pre-trained model averaged 0.933. These results highlight the limitations of generalized segmentation approaches. The PSN_adaptive_ framework achieved superior results, with DSC values progressively increasing as more fractions were incorporated into training. It achieved a DSC of 0.974 in the fourth fraction and a peak of 0.978 in the fifth fraction, demonstrating its ability to refine the segmentation accuracy effectively. The PSN_sequence_ framework also showed strong performance, achieving a DSC of 0.972 in the fourth fraction and 0.976 in the fifth fraction. While both PSN approaches performed comparably, PSN_adaptive_ demonstrated slightly better performance in the final fraction.

The deformed planning CT recorded an HD of 2.480 mm for the first fraction and 1.979 mm for the fifth fraction, showing modest improvements across fractions. The pre-trained model, however, exhibited consistently higher HD values, averaging 4.377 mm for the first fraction and increasing to 7.249 mm by the fifth fraction, reflecting its inability to adapt to patient-specific anatomy. The PSN_adaptive_ framework achieved the most substantial reductions in HD, with 1.649 mm for the fourth fraction and a low 1.681 mm for the fifth fraction, showcasing its capacity to accurately delineate complex anatomical structures. The PSN_sequence_ framework was comparable at the fourth fraction (1.642 mm) but higher at the fifth (2.198 mm), thus trailing PSN_adaptive_ in later fractions.

The deformed planning CT approach achieved an MSD of 0.540 mm for the fifth fraction, while the pre-trained model scored 1.662 mm, highlighting its significant inaccuracy in capturing surface boundaries. The PSN_adaptive_ framework significantly outperformed the baseline methods, with MSD values of 0.631 mm for the fourth fraction and 0.510 mm for the fifth fraction. The PSN_sequence_ framework achieved similar results, with an MSD of 0.546 mm for the fifth fraction, slightly trailing PSN_adaptive_ but still significantly outperforming the other methods.

The results described above clearly demonstrate the superior performance of the PSN framework in improving the segmentation accuracy for CTV compared to both deformed planning CT and the pre-trained model. While both PSN_adaptive_ and PSN_sequence_ showed substantial improvements, PSN_adaptive_ consistently outperformed PSN_sequence_, particularly in the later fractions. These findings validate the PSN framework’s potential as a robust and reliable tool for personalized treatment planning, with its ability to adapt to patient-specific CBCT data and achieve clinically accurate segmentation.

Moreover, the computational efficiency of the framework supports its clinical feasibility. The generalized model required approximately 24 hours for pre-training, while the PSN_adaptive_ training time increased linearly with the number of input fractions, ranging from 104 seconds (one fraction) to approximately 420 seconds (four fractions). The PSN_sequence_ framework maintained a constant per-fraction training time of ~100 seconds due to its stepwise sequential update strategy. Inference time for all methods averaged 110 seconds, including preprocessing and postprocessing steps, supporting potential integration into time-sensitive ART workflows.

The statistical analyses further supported these findings. For DSC the two-sided Wilcoxon signed-rank test comparing the pre-trained model with PSN_adaptive_ yielded W = 0, p = 0.0625 (Holm-adjusted p = 0.125, n = 5) in each of fractions 3–5; the pre-trained versus PSN_sequence_ contrast produced the same values. The corresponding contrasts for HD and MSD also returned p = 0.0625 (Holm p = 0.125). No statistically significant differences were detected between the deformable-registration method and any PSN variant (e.g., DSC, fraction 5, deform vs PSN_adaptive_: W = 6, p = 0.8125, p_Holm_ = 1.000; MSD, fraction 4: W = 5, p = 0.625, p_Holm_ = 1.000). Likewise, PSN_adaptive_ and PSN_sequence_ were indistinguishable across all metrics (largest observed difference: DSC, fraction 5, W = 3, p = 0.125, p_Holm_ = 0.313; MSD, fraction 5, W = 6, p = 0.3125, p_Holm_ = 0.625). All pre-train vs PSN contrasts exhibited very large effect sizes (|r| ≥ 0.8), confirming a substantial benefit from patient-specific fine-tuning despite the limited sample size (n = 5).

To further illustrate the consistency of the improvements at the individual-patient level, we provide per-patient DSC trajectories across fractions in S1 Fig; DSC was selected for this visualization because it clearly illustrates the patient-wise trends of adaptation between the pre-trained baseline and the PSN variants.

To complement the geometric evaluation, we compared dose–volume metrics against the physician‑defined CTV. Four absolute errors |ΔD_95_|, |ΔD_98_|, |ΔD_mean_| and |ΔD_2_| were computed for each fraction (Table 3; representative DVHs in S2 Fig). Paired two‑sided Wilcoxon signed‑rank tests (α = 0.05) with Holm adjustment and rank‑biserial effect sizes (95% bootstrap CI, 10 000 resamples) were applied across the five patients.

**Table 3:** Absolute deviations from physician-defined CTV dose (|ΔD_95_|, |ΔD_98_|, |ΔD_mean_|, |ΔD_2_|) across fractions.

Fraction	Method	| ΔD_95_|	| ΔD_98_|	|ΔD_mean_|	|ΔD_2_|
**2**^**nd**^ **fraction**	**DIR**	0.841 ± 0.386	0.842 ± 0.436	0.295 ± 0.258	0.018 ± 0.015
	**Pre-train**	1.992 ± 2.688‡	2.396 ± 3.162‡	0.646 ± 0.848‡	0.020 ± 0.012
	**PSN**_**adaptive**_	0.370 ± 0.373 †‡	0.360 ± 0.230 †‡	0.083 ± 0.136 †‡	0.003 ± 0.004†
	**PSN**_**sequence**_	0.370 ± 0.373 †‡	0.360 ± 0.230 †‡	0.083 ± 0.136 †‡	0.003 ± 0.004†
**3**^**rd**^ **fraction**	**DIR**	0.817 ± 0.660	0.749 ± 0.359	0.286 ± 0.416	0.016 ± 0.011
	**Pre-train**	2.586 ± 4.156‡	3.247 ± 5.037‡	0.717 ± 1.180‡	0.039 ± 0.035
	**PSN**_**adaptive**_	0.227 ± 0.212†	0.283 ± 0.169†	0.031 ± 0.024†	0.004 ± 0.003
	**PSN**_**sequence**_	0.439 ± 0.229 †‡	0.504 ± 0.439 †‡	0.089 ± 0.080 †‡	0.003 ± 0.003
**4**^**th**^ **fraction**	**DIR**	0.498 ± 0.285	0.462 ± 0.429	0.135 ± 0.166	0.014 ± 0.016
	**Pre-train**	2.408 ± 2.908‡	3.098 ± 3.844‡	0.651 ± 0.881‡	0.032 ± 0.042‡
	**PSN**_**adaptive**_	0.351 ± 0.239†	0.348 ± 0.235†	0.100 ± 0.108†	0.006 ± 0.008
	**PSN**_**sequence**_	0.278 ± 0.246†	0.294 ± 0.214†	0.089 ± 0.138†	0.003 ± 0.003
**5**^**th**^ **fraction**	**DIR**	0.589 ± 0.566	0.887 ± 0.364	0.215 ± 0.321	0.006 ± 0.006
	**Pre-train**	1.911 ± 2.496‡	2.607 ± 3.025‡	0.559 ± 0.655‡	0.037 ± 0.034‡
	**PSN**_**adaptive**_	0.274 ± 0.173†	0.389 ± 0.282†	0.074 ± 0.041†	0.012 ± 0.009
	**PSN**_**sequence**_	0.279 ± 0.338†	0.399 ± 0.596†	0.079 ± 0.069†	0.013 ± 0.014

Values are mean ± standard deviation over the five patients. †pHolm < 0.05 vs DIR; ‡pHolm < 0.05 vs Pre-train (Wilcoxon signed-rank).

Both PSN_adaptive_ and PSN_sequence_ yielded large, statistically significant improvements over the Pre‑train network for nearly all indices. For example, mean |ΔD_mean_| and |ΔD_2_| fell by ≥ 70% (adjusted p < 0.001, |r| ≥ 0.7), indicating better overall coverage and suppression of high‑dose hot spots. Coverage errors |ΔD_95_| and |ΔD_98_| were likewise reduced (adjusted p ≤ 0.0002).

When compared with DIR, improvements were more selective. PSN_adaptive_ significantly lowered |ΔD_95_| and |ΔD_98_| (adjusted p ≈ 0.0115), whereas changes in D_mean_ and D_2_ did not reach significance, suggesting DIR already captures bulk dose reasonably well. PSN_sequence_ showed a significant advantage over DIR mainly in |ΔD_98_|, reflecting its strength in reproducing the high‑dose tail. Direct PSN_adaptive_ vs PSN_sequence_ contrasts revealed no consistent significant differences, confirming comparable dosimetric performance.

Overall, these results mirror the geometric gains: patient‑specific fine‑tuning with the PSN framework produces dose distributions that are markedly closer to the physician reference than those from the generic model and, in selected metrics, than DIR, supporting the clinical value of both PSN approaches for adaptive, personalized radiotherapy planning.

### Visual evaluation of CTV segmentation results

**Fig 4:**
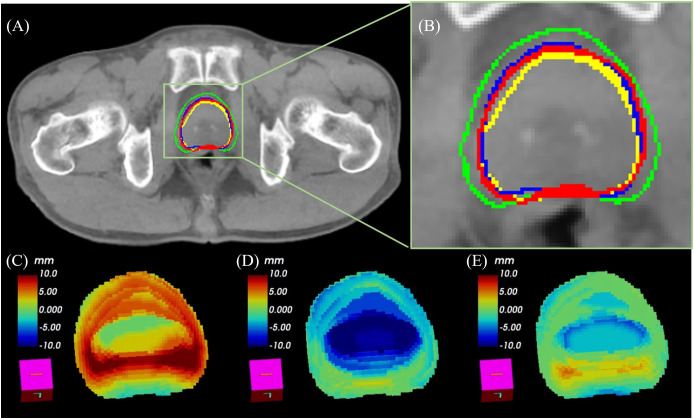
(a–b) Visual comparisons of CTV segmentation against reference contours (red), including outputs from the deformed planning CT (yellow), pre-trained model (green), and PSN_adaptive_ (blue). (a) Axial slice; (b) magnified view in a complex region. (c–e) 3D deviation maps between reference contours and segmentation results from the pre-trained model (c), deformed CT (d), and PSN_adaptive_ model trained on the 1^st^ fraction (e).

Fig 4(a) and (b) present visual comparisons of CTV segmentation results against reference contours manually annotated by a physician. The evaluated methods include the pre-trained model, deformed planning CT, and the PSN_adaptive_ framework.

In Fig 4 (a), the axial view demonstrates that contours generated by the pre-trained model (green) deviate notably from the reference, especially in regions with complex anatomical boundaries. The deformed planning CT (yellow) exhibits better conformity than the pre-trained model but still demonstrates inaccuracies in critical anatomical areas. Conversely, the PSN framework (blue) closely aligns with the physician-drawn contours (red), substantially reducing boundary discrepancies and enhancing the segmentation accuracy.

Notably, the PSN framework showed marked improvements in specific anatomical subregions, such as the apex and base of the prostate, where inter-fractional variability is typically higher and soft-tissue contrast is limited on CBCT. In the seminal vesicle region, especially in cases where it was partially included in the CTV, the PSN-based contours captured the elongated and variable shape more faithfully than both the pre-trained model and the deformed planning CT. These improvements were visually consistent across multiple test cases.

Fig 4(c-e) show the 3D deviation maps between the physician-drawn reference contours and segmentation results from each method. The deviation maps utilize color coding, with red and blue indicating positive and negative deviations, respectively.

(Fig 4 (c)) Pre-trained model vs. label: The map reveals considerable deviations throughout, underscoring the generalized model’s limitations in adapting to patient-specific anatomy. (Fig 4 (d)) Deformed planning CT vs. label: This method demonstrates improved alignment compared to the pre-trained model, but notable discrepancies persist, particularly at the boundary regions. (Fig 4 (e)) PSN framework vs. label: The deviation map displays minimal deviations, reflecting superior conformity with the reference contours and validating the effectiveness of the patient-specific PSN framework in achieving high segmentation accuracy

In particular, the deviation maps show that the largest improvements from PSN occurred at the anterior base and posterior apex, where CBCT quality often degrades and inter-observer variability is highest. The model’s ability to consistently match physician contours in these anatomically challenging regions underscores its clinical utility in adaptive radiotherapy.

## Discussion

This study significantly advances prostate CTV segmentation by integrating the Swin UNETR model within the PSN framework, a patient‑specific deep‑learning approach. Unlike generalized models, which often struggle to delineate individual patient anatomy accurately due to inter‑patient variability and limited adaptability to daily anatomical changes, PSN dynamically fine‑tunes segmentation using sequential CBCT data. Generalized deep‑learning models typically achieve suboptimal accuracy because of their fixed parameters and inability to incorporate sequential, patient‑specific data. Similarly, deformable registration methods, despite wide clinical use, frequently yield suboptimal contours owing to inherent inaccuracies in handling significant anatomical variations, as reported extensively in the radiotherapy literature.

Compared with previous studies on CBCT‑based online adaptive radiotherapy (oART), which primarily evaluated dosimetric improvements and workflow feasibility using the Ethos system but highlighted persistent limitations in contouring accuracy that required manual physician intervention, our study contributes a novel solution by directly addressing the challenge of precise auto‑segmentation in daily ART [3,25]. By integrating PSN with transformer‑based architectures, our approach demonstrates significant improvements in segmentation precision and establishes a robust foundation for efficient and reliable adaptive planning without frequent manual corrections. Visual assessments by experienced clinicians confirmed clinically plausible segmentation, showing improvements over both Ethos‑generated deformation‑based contours and baseline Swin UNETR segmentations.

In this study, we quantitatively evaluated PSN_adaptive_ and PSN_sequence_ using DSC, HD, and MSD standard segmentation metrics that, although not directly used in clinical decision‑making, serve as important indicators of technical accuracy. The achieved DSCs exceeded 0.97, well above the commonly cited clinical acceptability threshold of 0.90 for prostate targets [26] and surpassing the 0.95 level often interpreted as highly reliable [26,27]. Regarding HD, values below 3 mm have been suggested as a spatial‑accuracy requirement for clinical prostate contouring [28], and both PSN variants consistently met this benchmark. Similarly, MSD values remained below 1 mm, with values under 0.6 mm reflecting excellent conformity with expert contours [29]. These results not only indicate technical excellence but also point to strong potential for clinical adoption with minimal adaptation.

To further validate the consistency and generalizability of our findings, we additionally evaluated PSN using Dynamic UNet, a widely used, publicly benchmarked segmentation backbone [30,31]. Results (S2 and S3 Table in S1 File) paralleled those obtained with Swin UNETR across all metrics, reinforcing the architecture‑independent benefits of PSN in adaptive prostate segmentation.

Notably, recent studies employing personalized deep‑learning strategies akin to PSN have consistently shown superior segmentation accuracy versus generalized methods and deformable registration in prostate cancer radiotherapy [13,32,33]. While earlier work relied mainly on MRI‑based ART data, our study demonstrates that accurate patient‑specific segmentation is also achievable in CBCT workflows despite the modality’s lower soft‑tissue contrast. A key distinguishing feature of PSN is its formalization of continuous, sequential adaptation: unlike static patient‑specific models, PSN is updated using the complete series of daily CBCTs, making it uniquely suited for online ART workflows and marking a conceptual advance over previous personalization strategies [34].

Clinically, PSN offers substantial workflow benefits by automating adaptive segmentation, reducing physician workload, and improving reproducibility. Its fast incremental training enables integration into routine practice without disrupting ART schedules. Similar to the Ethos intelligent‑optimization engine (IOE) that has demonstrated planning efficiency in real‑world ART [35], PSN facilitates rapid adaptation and enhances confidence in daily contours, particularly in anatomically complex regions where deformable registration struggles.

Although Wilcoxon tests did not show statistical significance (adjusted p > 0.05) for most metrics, particularly in terms of DSC, PSN offers practical and conceptual advantages that extend beyond mean segmentation accuracy. DIR relies on predefined deformation fields and may produce spatially inconsistent errors in anatomically irregular or low-contrast regions. In contrast, PSN enables dynamic, patient-specific adaptation without manual tuning or reliance on registration assumptions. By operating in an end-to-end fashion and directly optimizing ground-truth masks, PSN avoids potential image-warping artifacts and reduces error propagation. The consistency and reduced inter-fraction variability observed across patients (S1 Fig) further support its robustness, indicating stable improvements rather than outlier-driven gains. Moreover, PSN_sequence_ offers constant and predictable per-step training time, while PSN_adaptive_ enables deeper personalization as more fractions are assimilated, providing clinicians with control over the latency–performance trade-off. These properties collectively position PSN as a reliable and efficient alternative, or complement, to DIR-based workflows, especially in anatomically complex or time-constrained adaptive radiotherapy settings.

Beyond target‑volume segmentation, the PSN framework also shows a promise for OARs such as bladder and rectum, which typically exhibit substantial inter-fraction anatomical and volumetric variation during prostate radiotherapy. These variations are often driven by physiological factors, for example, variable bladder filling or rectal gas, rather than stable patient-specific anatomy, and they frequently challenge the accuracy of deformable registration. In contrast, PSN’s sequential learning mechanism enables dynamic adaptation by leveraging daily CBCT data, allowing it to model evolving organ shapes over time and potentially improving robustness in regions where registration often fails. Recent CBCT-based studies have demonstrated that deep learning can achieve accurate segmentation of these OARs despite the high variability of the organs: Fu et al. reported mean DSCs of 0.96 and 0.93 for the bladder and the rectum, respectively, using CBCT and synthetic MR [7]; Léger et al. achieved 0.874 and 0.814 with cross-domain augmentation [36]; and Radici et al. confirmed that deep learning-generated contours are clinically acceptable and require minimal manual revision [37]. Based on these findings, PSN may further enhance OAR segmentation by learning patient-specific physiological patterns rather than relying on the static anatomical assumptions, offering the potential to reduce manual contour edits and streamline adaptive workflows in online ART.

Despite these encouraging results, our study has several limitations that inform future directions. First, the analysis relied on a relatively small, single‑institution cohort (26 patients, 119 fractions in total; evaluations on five patients, 25 fractions) acquired on one CBCT platform (Ethos), which may restrict generalizability. Second, PSN performance depends on stable CBCT image quality and introduces additional computational overhead that could challenge clinical throughput. To address these issues, we are (i) forming multi‑institutional collaborations to enlarge and diversify the dataset, (ii) testing PSN robustness under variable imaging protocols and scanner types, (iii) optimizing code and hardware for real‑time execution, and (iv) exploring reinforcement‑learning‑based online adaptation. Prospective studies will also measure contour‑editing time savings and dosimetry impact to establish clinically meaningful end‑points, paving the way for PSN deployment across diverse adaptive‑radiotherapy platforms and anatomical sites [25,32–35,38–40].

## Conclusion

This study demonstrates that the PSN framework enables highly accurate, patient-specific segmentation for CBCT-guided adaptive radiotherapy in prostate cancer, achieving clinically acceptable performance (mean DSC > 0.97, HD < 3 mm) and substantially reducing manual contour-editing requirements compared with both generalized deep learning models and deformable image registration. By supporting rapid, incremental model updates using daily imaging data, PSN integrates seamlessly into online adaptive workflows and offers a scalable foundation for broader deployment across diverse anatomical sites and imaging platforms.

## Supporting information

S1 File Supporting information. This file includes S1 Appendix (Equations for evaluation metrics), S1 Table (Patient Characteristics), S2 Table (Average DSC, HD, and MSD for the PSNadaptive approach using Dynamic Unet), and S3 Table (Average DSC, HD, and MSD for the PSNsequence approach using Dynamic Unet).
(PDF)

S2 File Minimal data set. This file contains the underlying raw data for the five patients evaluated in this study. It includes the individual values for all geometric metrics (DSC, HD, MSD) and dosimetric metrics (|ΔD_95_|, |ΔD_98_|, |ΔD_mean_|, |ΔD_2_|) which were used to calculate the summary statistics presented in the manuscript’s tables.
(XLSX)

S1 Fig Patient-wise DSC across fractions. This figure illustrates the per-patient DSC trajectory, comparing the pre-trained model with the PSN_adaptive_ and PSN_sequence_ variants over five treatment fractions.
(JPG)

S2 Fig DVH for a representative patient. This figure shows a representative DVH for the fifth treatment fraction of a single patient, comparing the dose distribution for contours generated by the physician (reference), DIR, pre-trained model, PSN_adaptive_, and PSN_sequence_ methods.
(JPG)
