# Supplementary material for: Improving segmentation precision in prostate cancer adaptive radiation therapy with a patient-specific network
Source: PLoS One. 2025 Sep 19;20(9):e0332603. doi: 10.1371/journal.pone.0332603 (PMC12448962; doi:10.1371/journal.pone.0332603)
Supplement: S1 File — This file includes S1 Appendix (Equations for evaluation metrics), S1 Table (Patient Characteristics), S2 Table (Average DSC, HD, and MSD for the PSNadaptive approach using Dynamic Unet), and S3 Table (Average DSC, HD, and MSD for the PSNsequence approach using Dynamic Unet). (PDF) [file pone.0332603.s001.pdf]

S1 Appendix

Eq. 1: Dice Similarity Coefficient (DSC)

The DSC is a statistical measure evaluating the overlap between two regions, particularly suitable for image segmentation tasks. It quantifies the agreement between the predicted segmentation (X) and the ground truth (Y) with values ranging from 0 (no overlap) to 1 (perfect overlap):

$$DSC = \frac{(2\times|X\cap Y|)}{(|X|+|Y|)} \qquad \text{(Eq. 1)}$$

where $|X\cap Y|$ is the intersection of the predicted and ground truth volumes, and $|X|$ and $|Y|$ are the corresponding sizes.

Eq. 2: 95th Percentile Hausdorff Distance (HD)

The 95th percentile HD measures the dissimilarity between two sets by capturing the largest distance from a point in one set to the closest point in the other set. HD is particularly useful in medical imaging for identifying outliers and quantifying boundary discrepancies:

$$H(X,Y) = max(Sup_{x\in X} inf_{y\in Y} d(x,y), Sup_{y\in Y} inf_{x\in X} d(x,y)) \qquad \text{(Eq. 2)}$$

where, $\boldsymbol{d(x,y)}$ represents the distance between points x and y, and Sup and inf represent the supremum (least upper bound) and infimum (greatest lower bound), respectively.

Eq. 3: Mean Surface Distance (MSD)

The MSD calculates the average distance between corresponding points on the surfaces of two segmented regions, providing an overall indication of segmentation accuracy:

$$MSD = \frac{1}{N}\sum_{i=1}^{N} d(x_i, y_i) \qquad \text{(Eq. 3)}$$

where N is the total number of corresponding point pairs, and $d(x_i, y_i)$ is the distance between a point x_i on one surface and its corresponding point y_i on the other.

S1 Table. Patient Characteristics

Age (years)	Stage	Gleason Score	Initial PSA (ng/mL)	Androgen Deprivation Therapy (ADT)	Target Volume	Prescription
83	cT2cN0M0	4+4	5.8	under ADT	prostate	36.5 Gy in 5 fractions
86	cT1cN0M0	3+4	5.9	under ADT	prostate	36.5 Gy in 5 fractions
84	cT2cN0M0	4+5	3.4	under ADT	prostate	36.5 Gy in 5 fractions
69	cT3aN0M0	3+3	11.5	no ADT	prostate	36.5 Gy in 5 fractions
76	cT3aN1M1	4+4	115	under ADT	prostate	36.5 Gy in 5 fractions
77	cT2bN0M0	3+4	5.6	under ADT	prostate	36.5 Gy in 5 fractions
68	cT2cN0M0	3+3	10.2	under ADT	prostate	36.5 Gy in 5 fractions
68	cT2bN0M0	3+5	17.2	under ADT	prostate	36.5 Gy in 5 fractions
84	cT2cN0M0	3+4	7.9	under ADT	prostate	36.5 Gy in 5 fractions
78	cT3aN0M0	4+3	8.9	under ADT	prostate	36.5 Gy in 5 fractions
63	cT2cN0M0	4+3	12	under ADT	prostate	36.5 Gy in 5 fractions
84	cT2bN0M0	4+5	5.5	under ADT	prostate	36.5 Gy in 5 fractions
77	cT2aN0M0	3+3	11.9	under ADT	prostate	36.5 Gy in 5 fractions
84	cT3aN0M0	3+4	23	under ADT	prostate	36.5 Gy in 5 fractions
80	cT3aN0M0	4+3	2.6	under ADT	prostate/seminal vesicles	36.5 Gy in 5 fractions
63	cT2cN0M0	3+4	6	under ADT	prostate/seminal vesicles	46 Gy in 23 fractions + 24 Gy in 12 fractions
63	cT3aN0M0	4+3	30	no ADT	whole pelvis/prostate	46 Gy in 23 fractions + 18 Gy in 4 fractions
68	cT3aN0M0	3+4	5.7	under ADT	whole pelvis/prostate	46 Gy in 23 fractions + 18 Gy in 3 fractions
72	cT3aN0M0	4+3	5.1	no ADT	whole pelvis/prostate	46 Gy in 23 fractions + 18 Gy in 3 fractions
74	cT3bN0M0	3+4	8.9	under ADT	whole pelvis/prostate	46 Gy in 23 fractions + 18 Gy in 4 fractions
76	cT3aN0M0	3+4	138	under ADT	whole pelvis/prostate	46 Gy in 23 fractions + 18 Gy in 4 fractions
80	cT3aN0M0	3+4	12.5	no ADT	whole pelvis/prostate	50 Gy in 25 fractions + 30 Gy in 15 fractions
80	cT3aN0M0	4+4	29.9	under ADT	whole pelvis/prostate	46 Gy in 23 fractions + 18 Gy in 4 fractions
83	cT2cN0M0	4+4	10.2	under ADT	whole pelvis/prostate	46 Gy in 23 fractions + 34 Gy in 17 fractions
83	cT3aN0M0	4+5	39.6	under ADT	whole pelvis/prostate	46 Gy in 23 fractions + 18 Gy in 4 fractions
84	cT3aN0M0	4+4	6.6	under ADT	whole pelvis/prostate	46 Gy in 23 fractions + 18 Gy in 3 fractions

S2 Table. Average DSC, HD, and MSD for the Deform from ETHOS, the pre-trained Dynamic Unet model, and the $PSN_{adaptive}$ approach using Dynamic Unet, along with their standard deviations, as illustrated in Fig 2A

		**Deform**	**Pre-train**	**PSN trained on 1 fx**	**PSN trained on 1-2 fx**	**PSN trained on 1-3 fx**	**PSN trained on 1-4 fx**
**CTV DSC**	**1st**	0.961 ± 0.020	0.927 ± 0.025				
	**2nd**	0.963 ± 0.015	0.930 ± 0.031	0.967 ± 0.012			
	**3rd**	0.970 ± 0.014	0.930 ± 0.043	0.970 ± 0.014	0.971 ± 0.010		
	**4th**	0.974 ± 0.012	0.935 ± 0.039	0.966 ± 0.011	0.970 ± 0.008	0.975 ± 0.009	
	**5th**	0.977 ± 0.004	0.934 ± 0.030	0.971 ± 0.011	0.974 ± 0.008	0.976 ± 0.008	0.978 ± 0.009
**CTV HD**	**1st**	2.480 ± 0.560	4.005 ± 0.674				
	**2nd**	2.694 ± 0.822	4.024 ± 1.004	2.159 ± 0.414			
	**3rd**	2.727 ± 1.868	4.154 ± 1.561	1.941 ± 0.888	1.759 ± 0.362		
	**4th**	1.860 ± 0.418	3.527 ± 1.397	2.021 ± 0.627	1.930 ± 0.306	1.659 ± 0.248	
	**5th**	1.979 ± 0.597	3.835 ± 0.784	2.014 ± 0.824	1.881 ± 0.760	2.049 ± 1.120	1.943 ± 1.425
**CTV MSD**	**1st**	0.840 ± 0.290	1.517 ± 0.453				
	**2nd**	0.828 ± 0.166	1.494 ± 0.624	0.760 ± 0.116			
	**3rd**	0.730 ± 0.328	1.521 ± 0.998	0.704 ± 0.214	0.699 ± 0.130		
	**4th**	0.599 ± 0.134	1.476 ± 0.833	0.831 ± 0.139	0.739 ± 0.077	0.610 ± 0.126	
	**5th**	0.540 ± 0.083	1.444 ± 0.669	0.690 ± 0.132	0.625 ± 0.112	0.546 ± 0.055	0.501 ± 0.065

S3 Table. Average DSC, HD, and MSD for the Deform from ETHOS, the pre-trained Dynamic Unet model, and the $PSN_{sequence}$ approach using Dynamic Unet, along with their standard deviations, as illustrated in Fig 2B

		Deform	Pre-train	PSN trained on 1 fx	PSN trained on 1-2 fx	PSN trained on 1-3 fx	PSN trained on 1-4 fx
**CTV DSC**	**1st**	0.961 ± 0.020	0.927 ± 0.025				
	**2nd**	0.963 ± 0.015	0.930 ± 0.031	0.967 ± 0.012			
	**3rd**	0.970 ± 0.014	0.930 ± 0.043	0.970 ± 0.014	0.971 ± 0.009		
	**4th**	0.974 ± 0.012	0.935 ± 0.039	0.966 ± 0.011	0.971 ± 0.009	0.971 ± 0.008	
	**5th**	0.977 ± 0.004	0.934 ± 0.030	0.971 ± 0.011	0.973 ± 0.008	0.975 ± 0.008	0.977 ± 0.007
**CTV HD**	**1st**	2.480 ± 0.560	4.005 ± 0.674				
	**2nd**	2.694 ± 0.822	4.024 ± 1.004	2.159 ± 0.414			
	**3rd**	2.727 ± 1.870	4.154 ± 1.561	1.941 ± 0.888	1.903 ± 0.474		
	**4th**	1.860 ± 0.418	3.527 ± 1.397	2.021 ± 0.627	1.887 ± 0.227	1.759 ± 0.362	
	**5th**	1.979 ± 0.597	3.835 ± 0.784	2.014 ± 0.824	1.945 ± 0.724	1.995 ± 1.129	1.970 ± 0.949
**CTV MSD**	**1st**	0.840 ± 0.290	1.517 ± 0.453				
	**2nd**	0.828 ± 0.166	1.494 ± 0.624	0.760 ± 0.116			
	**3rd**	0.730 ± 0.328	1.521 ± 0.998	0.704 ± 0.214	0.689 ± 0.138		
	**4th**	0.599 ± 0.134	1.476 ± 0.833	0.831 ± 0.139	0.705 ± 0.083	0.690 ± 0.109	
	**5th**	0.540 ± 0.083	1.444 ± 0.669	0.690 ± 0.132	0.631 ± 0.071	0.580 ± 0.030	0.541 ± 0.054
