# Supplementary figures and images for: Improving segmentation precision in prostate cancer adaptive radiation therapy with a patient-specific network
Source: PLoS One. 2025 Sep 19;20(9):e0332603. doi: 10.1371/journal.pone.0332603 (PMC12448962; doi:10.1371/journal.pone.0332603)

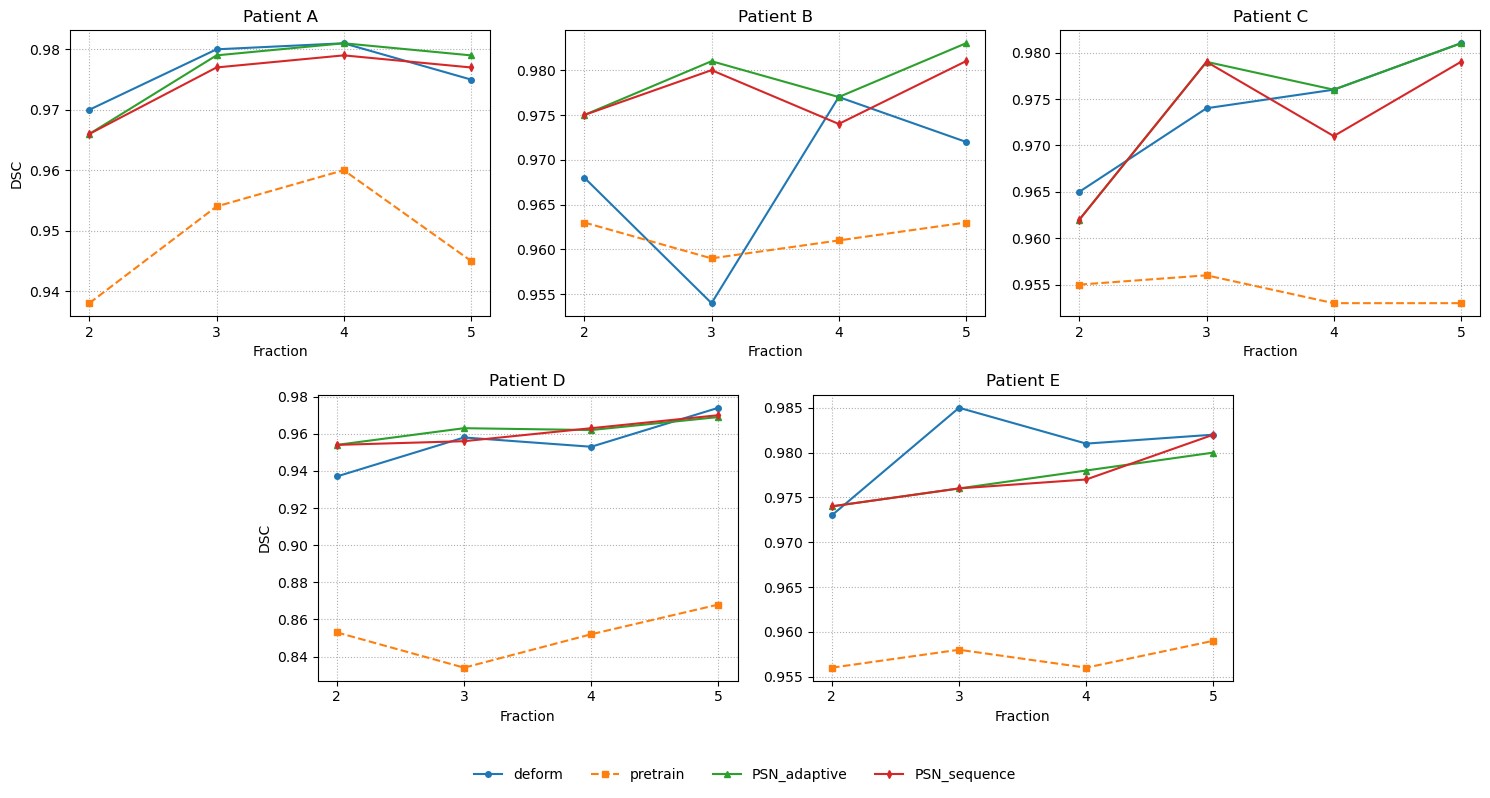

Supplement: S1 Fig — This figure illustrates the per-patient DSC trajectory, comparing the pre-trained model with the PSNadaptive and PSNsequence variants over five treatment fractions. (JPG) [file pone.0332603.s003.jpg]

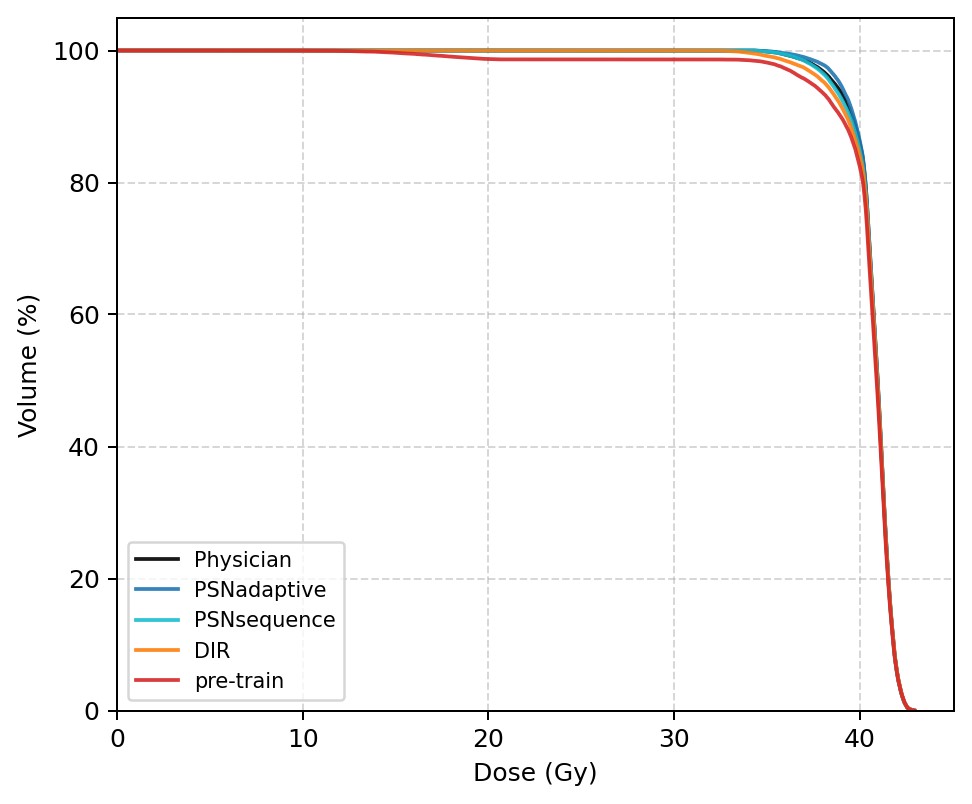

Supplement: S2 Fig — This figure shows a representative DVH for the fifth treatment fraction of a single patient, comparing the dose distribution for contours generated by the physician (reference), DIR, pre-trained model, PSNadaptive, and PSNsequence methods. (JPG) [file pone.0332603.s004.jpg]
